# The Open Translational Science in Schizophrenia (OPTICS) project: an open-science project bringing together Janssen clinical trial and NIMH data

**DOI:** 10.1038/s41537-018-0055-7

**Published:** 2018-06-27

**Authors:** Marsha A. Wilcox, Adam J. Savitz, Anjené M. Addington, Gary S. Gray, Eva C. Guinan, John W. Jackson, Thomas Lehner, Sharon-Lise Normand, Hardeep Ranu, Geetha Senthil, Jake Spertus, Linda Valeri, Joseph S. Ross

**Affiliations:** 1 Janssen Pharmaceutical Research and Development, Titusville, NJ USA; 2 Genomics Research Branch, National Institute of Mental Health in Bethesda, Bethesda, MD USA; 3 Harvard Catalyst|The Harvard Clinical and Translational Science Center, Harvard Medical School, Boston, MA USA; 4 Department of Radiation Oncology, Dana-Farber Cancer Institute and Harvard Medical School, Boston, MA USA; 5 Department of Epidemiology, Johns Hopkins Bloomberg School of Public Health, Baltimore, MD USA; 6 Department of Epidemiology, Harvard T.H. Chan School of Public Health, Boston, MA USA; 7 Office of Genomics Research Coordination, National Institute of Mental Health, Bethesda, USA; 8 Department of Health Care Policy (Biostatistics), Harvard Medical School, Boston, MA USA; 9 Department of Psychiatry, Harvard Medical School, Boston, MA USA; 10 Laboratory for Psychiatric Biostatistics, McLean Hospital, Belmont, MA USA; 11 Section of General Internal Medicine and the National Clinician Scholars Program, Department of Medicine, Yale University School of Medicine, New Haven, CT USA; 12 Department of Health Policy and Management, Yale University School of Public Health, New Haven, CT USA; 13 The Center for Outcomes Research and Evaluation, Yale–New Haven Hospital, New Haven, CT USA

## Abstract

Clinical trial data are the gold standard for evaluating pharmaceutical safety and efficacy. There is an ethical and scientific imperative for transparency and data sharing to confirm published results and generate new knowledge. The Open Translational Science in Schizophrenia (OPTICS) Project was an open-science initiative aggregating Janssen clinical trial and NIH/NIMH data from real-world studies and trials in schizophrenia. The project aims were to show the value of using shared data to examine: therapeutic safety and efficacy; disease etiologies and course; and methods development. The success of project investigators was due to collaboration from project applications through analyses, with support from the Harvard Catalyst. Project work was independent of Janssen; all intellectual property was dedicated to the public. Efforts such as this are necessary to gain deeper insights into the biology of disease, foster collaboration, and to achieve the goal of developing better treatments, reducing the overall public health burden of devastating brain diseases.

## Introduction

### Data sharing

Clinical trial data are the gold standard for evaluating pharmaceutical safety and efficacy. Until recently, these data have been sequestered within companies, sponsors of much of the clinical research conducted on pharmaceutical products. There is an ethical and scientific imperative for transparency and sharing of these data to confirm published results and generate new knowledge.^1–4^ Transparency has become the new standard in pharmaceutical and medical device science.^5,6^ Recent initiatives have highlighted the need for transparency and sharing, among these are The Institute of Medicine’s report, “Sharing Clinical Trial Data: Maximizing Benefits, Minimizing Risks” and guidance from international organizations and industry.

### The Open Translational Science in Schizophrenia (OPTICS) Project, an overview

**Fig. 1:**
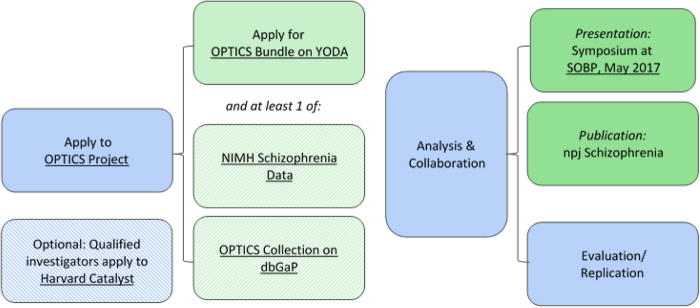
The OPTICS Project Process

Janssen and a group of leading research organizations launched the OPTICS Project in 2015. This industry-academic-government collaboration aggregated Janssen clinical trial and federally-funded data about schizophrenia. Researchers from diverse disciplines collaborated in an open-science environment addressing essential questions about schizophrenia, therapeutic safety and efficacy, and methods development. All IP generated has been dedicated to the public and is free for all to use. In this effort, data about the disorder and clinical trials of therapies were made available to researchers in one place. Our goal was to advance scientific knowledge about the disease, foster collaboration, and create new models for conducting research. Figure 1 depicts this process.

### The Yale Open Data Access (YODA) project

The YODA Project was founded in 2011 to promote data sharing among the scientific community and develop a platform that could be used as a means of responsible data sharing.^7^ Through partnerships with Johnson & Johnson and Medtronic, Inc., the YODA Project established policies to make shared data available to investigators, including de-identified participant-level trial data, comprehensive trial reports, and supportive documentation and meta-data. Data are currently available for more than 250 clinical trials of different psychiatric conditions, as well as non-psychiatric conditions, to facilitate research that may advance science or lead to improvements in health and health care delivery. Janssen data for the OPTICS Project were made available through the YODA Project.

### National Institute of Mental Health (NIMH) perspective

NIMH has long been a strong proponent of endeavors, such as the OPTICS Project, that encourage researchers to leverage shared resources for broader scientific discovery. The NIMH Repository and Genomics Resource, established under the 1989 NIMH Human Genetics Initiative, currently shares clinical data and samples from over 200,000 subjects spanning many psychiatric disorders and populations. Additionally, NRGR and Database of Genotypes and Phenotypes (dbGaP) together provide access to genome-wide data from over 87,000 subjects from psychiatric genetics studies. NIMH further provides access to a variety of clinical and data through the recently established NIMH Data Archives. NIMH is eager to foster collaborations with industry partners that will leverage clinical trial data (e.g., genomic screens) that is not broadly available to enhance the secondary analyses done using our publicly available datasets. Such collaborations could provide many opportunities for discovery and may expedite identification and prioritization of potential molecular targets for therapeutic development for psychiatric disorders.

### Harvard Catalyst (HC) perspective

The Harvard Catalyst| The Harvard Clinical and Translational Science Center^8^ and OPTICS Project collaboration began with the “ReSourcing Big Data”^9^ symposium on collaborative data reuse/sharing. One outcome of the symposium was a joint pilot grant program to support analysis of OPTICS data. Grant awardees were selected on a competitive basis from applicants throughout Harvard Medical School and affiliates and supported for 12 months. The HC-OPTICS pilot grant program was successful as measured by the insights produced, the quality of the research and analyses completed, the number of analytic tools developed, publications generated, and the potential for subsequent funding. These outcomes are described in detail elsewhere in this issue. Moreover, several observations from this pilot grant project may be relevant to future data reuse efforts (Table 1).

**Table 1:** Data reuse success factors

Success factor	Observation
Education	Provided good description of opportunity, including face-to-face Q&A
	Demonstrated access to and use of data set
Data set	Should be complete, consistent, well curated, and readily accessible
	Lack of familiarity with format or non-standard format adds challenge
Communication	Frequent, both between teams and data set curators and among teams
	Designed to foster cross-team collaboration, learning, and problem solving
Data Use Agreements	Completed agreement is a limiting factor. Timing and content varies among institutions

The HC-OPTICS pilot grant project demonstrated the potential for highly productive reuse of existing clinical trials data sets.

### Lessons learned

This pilot was designed to inform future efforts of this kind. The most important lesson learned was the importance of a collaborative environment; on-going dialogue with other investigators and the data holders contributed to successfully completing analyses and producing manuscripts.

From a pragmatic perspective, it is important that one never underestimate the time required to gain access to data, which includes IRB approval and especially institutional signing of Data Use Agreements, as well as the time required for external investigators to gain facility with the shared data. To address the former, Data Use Agreements should be standardized and pursued as soon as possible. To address the latter, data sharing platforms should ensure that data descriptions, dictionaries, protocols, and statistical analysis plans are available early, preferably during the application period, and that the computing environment is up-to-date, including statistical software. Finally, we would recommend that external investigators reproduce sample characteristics and primary results prior to pursuing their independent research analyses to identify possible discrepancies or data misunderstandings as soon as possible. All parties should anticipate frequent communications (e.g., questions about the data, the statistical software, the sponsors analyses), which foster collaboration.

The following manuscripts were the result of work pursued within OPTICS Project:

*“Risk of weight gain for specific antipsychotic drugs: A Bayesian network meta-analysis of individual participant level clinical trial data”* by Jacob Spertus, Marcela Horvitz-Lennon, Haley Abing, and Sharon-Lise Normand.

*“The role of PANSS symptoms and adverse events in explaining the effects of Paliperidone on social functioning: a causal mediation analysis approach”* by Xue Zou, Yiwen Zhu, John W. Jackson, Andrea Bellavia, Garrett Fitzmaurice, Franca Centorrino, and Linda Valeri.

## Conclusions

The OPTICS Project was a collaboration in which both observational and interventional data about schizophrenia were made available to researchers in an open-science effort. The goal was to provide a forum for translational science in schizophrenia research. Aggregating and sharing these data enabled researchers to address questions about the disease, therapies, and analytic methods in ways not before possible. While the value of sharing clinical trial data on its own is significant, this project also provided a unique opportunity for collaborative partnerships.

The OPTICS Project is representative of a new paradigm in scientific research which we hope will lead to more collaboration and data sharing among industry and the broader research community. Such cooperative efforts are necessary to gain deeper insights into the biology of disease, to achieve the goal of developing better treatments and reducing the overall public health burden of devastating brain diseases.

### Data availability

Janssen clinical trials available at the YODA Project: http://yoda.yale.edu/multiple-ncts-optics-trial-bundle

NIMH: https://nimhgenetics.org/available_data/schizophrenia/

dbGaP: https://www.ncbi.nlm.nih.gov/projects/gap/cgi-bin/collection.cgi?study_id=phs000887.v1.p1
